# Aquabis­(6-bromo­picolinato-κ^2^ *N*,*O*)copper(II)

**DOI:** 10.1107/S1600536808041536

**Published:** 2008-12-10

**Authors:** Fei-Long Hu, Zhuang Yue, Mi Yan, Wei-Qiang Luo, Xian-Hong Yin

**Affiliations:** a College of Chemistry and Ecological Engineering, Guangxi University for Nationalities, Nanning 530006, People’s Republic of China

## Abstract

In the title compound, [Cu(C_6_H_3_BrNO_2_)_2_(H_2_O)], the Cu atom adopts a distorted trigonal-bipyramidal coordination arising from two *N*,*O*-bidentate ligands and a water mol­ecule, with one N atom in an axial site and the other in an equatorial site. The dihedral angle between the pyridine ring planes is 67.6 (2)°. In the crystal, O—H⋯O hydrogen bonds result in chains propagating in [100].

## Related literature

For background, see: Mann *et al.* (1992 ▶).
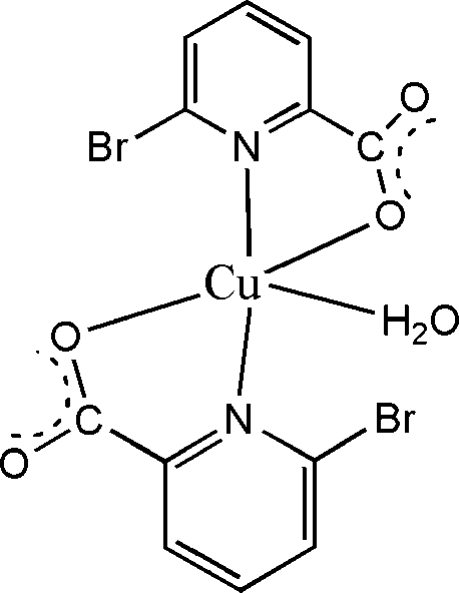


## Experimental

### 

#### Crystal data

[Cu(C_6_H_3_BrNO_2_)_2_(H_2_O)]
*M* *_r_* = 483.56
Triclinic, 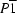

*a* = 6.9447 (8) Å
*b* = 9.1350 (10) Å
*c* = 11.4510 (13) Å
α = 86.741 (2)°
β = 84.056 (2)°
γ = 76.728 (1)°
*V* = 702.84 (14) Å^3^
*Z* = 2
Mo *K*α radiation
μ = 7.26 mm^−1^
*T* = 298 (2) K
0.18 × 0.14 × 0.08 mm

#### Data collection

Siemens SMART CCD diffractometer
Absorption correction: multi-scan (*SADABS*; Sheldrick, 1996 ▶) *T*_min_ = 0.354, *T*_max_ = 0.594 (expected range = 0.333–0.559)
3669 measured reflections
2435 independent reflections
2137 reflections with *I* > 2σ(*I*)
*R*_int_ = 0.018

#### Refinement

*R*[*F* ^2^ > 2σ(*F* ^2^)] = 0.032
*wR*(*F* ^2^) = 0.082
*S* = 1.02
2435 reflections
199 parameters
H-atom parameters constrained
Δρ_max_ = 0.61 e Å^−3^
Δρ_min_ = −0.59 e Å^−3^

### 

Data collection: *SMART* (Siemens, 1996 ▶); cell refinement: *SAINT* (Siemens, 1996 ▶); data reduction: *SAINT*; program(s) used to solve structure: *SHELXS97* (Sheldrick, 2008 ▶); program(s) used to refine structure: *SHELXL97* (Sheldrick, 2008 ▶); molecular graphics: *SHELXTL* (Sheldrick, 2008 ▶); software used to prepare material for publication: *SHELXTL*.

## Supplementary Material

Crystal structure: contains datablocks I, global. DOI: 10.1107/S1600536808041536/hb2875sup1.cif

Structure factors: contains datablocks I. DOI: 10.1107/S1600536808041536/hb2875Isup2.hkl

Additional supplementary materials:  crystallographic information; 3D view; checkCIF report

## Figures and Tables

**Table 1:** Selected bond lengths (Å)

Cu1—O1	1.912 (3)
Cu1—N2	1.985 (3)
Cu1—O5	2.022 (3)
Cu1—O3	2.072 (3)
Cu1—N1	2.148 (3)

**Table 2:** Hydrogen-bond geometry (Å, °)

*D*—H⋯*A*	*D*—H	H⋯*A*	*D*⋯*A*	*D*—H⋯*A*
O5—H5*A*⋯O1^i^	0.85	1.93	2.765 (4)	168
O5—H5*B*⋯O4^ii^	0.85	1.90	2.743 (4)	169

Symmetry codes: (i) 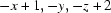; (ii) 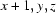.

**Fig. 1.**
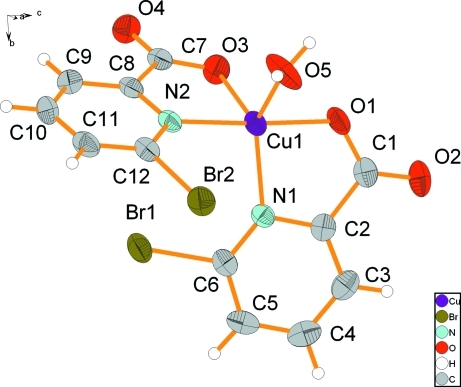
The molecular structure of (I) showing 30% probability displacement ellipsoids for the non-hydrogen atoms.

**Fig. 2.**
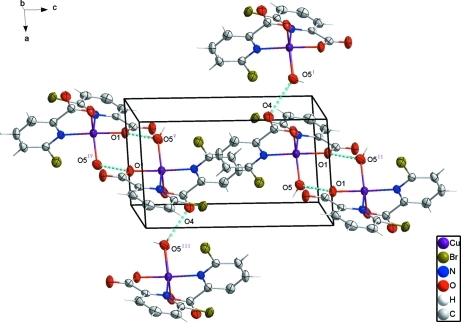
Part of the hydrogen bonding network, the hydrogen bonded interactions are showing as dashed lines. [symmetry codes: (I) -1+x, y, z; (II) 1-x, -y, 2-z; (III) 2-x, 1-y, 1-z; (IV) x, 1+y, -1+z; (V) 1-x, 1-y, 1-z.]

## supplementary crystallographic information

### Comment

The chemical and pharmacological properties of pyridine derivatives have been investigated extensively, owing to their chelating ability with metal ions and their potentially beneficial chemical and biological activities (e.g. Mann *et al.*, 1992). As part of our studies on the synthesis and characterization of these compounds, we report here the synthesis and crystal structure of the title compound, (I), (Fig. 1).

The copper centre in (I) adopts distorted trigonal biyramid coordination geometry by being coordinated with two nitrogen atoms from the pyridine rings and three oxygen atoms from the ligands (Table 1). The dihedral angle of the two pyridine rings is 67.6 (2)°.

Analysis of the crystal packing of the title compound reveals the existence of intermolecular O—H···O hydrogen bonds between the carboxyl oxygen atoms and coordinated water molecule (Fig. 2), forming a one-dimensional chain parallel to the a-axis. The coordinated water molecule acts as a hydrogen-bond donor towards O1 and O4 of the adjacent complexes (Table 2), the carboxylate group that acts as an H bond acceptor towards the O5 via both of its O atoms O4 and O3 exhibits a delocalized π system with nearly identical C—O distances.

### Experimental

1 mmol (200.9 mg) of 6-bromopicolinic acid was added to 0.5 mmol (132 mg) of CuCl_2_ in 10 ml of anhydrous alcohol.  The suspension was stirred for *ca* 4 h and filtered. After keeping the filtrate in air for one week, blue blocks of (I) precipitated. The crystals were isolated, washed with alcohol three times and dried in a vacuum desiccator using silica gel (Yield 75%). Elemental analysis: found C, 29.79; H, 1.68; N, 5.78; calc. for C_12_H_8_N_2_Br_3_O_5_Cu: C, 29.81; H, 1.67; N, 5.79.

### Refinement

The H atoms were positoned geometrically (C—H = 0.93Å, O—H = 0.85Å) and refined as riding with *U*_iso_(H) = 1.2*U*_eq_(C) or 1.5*U*_eq_(O).

### Crystal data

**Table:**

[Cu(C_6_H_3_BrNO_2_)_2_(H_2_O)]	*Z* = 2
*M**_r_* = 483.56	*F*(000) = 466
Triclinic, *P*1	*D*_x_ = 2.285 Mg m^−^^3^
Hall symbol: -P 1	Mo *K*α radiation, λ = 0.71073 Å
*a* = 6.9447 (8) Å	Cell parameters from 2146 reflections
*b* = 9.135 (1) Å	θ = 2.3–28.1°
*c* = 11.4510 (13) Å	µ = 7.26 mm^−^^1^
α = 86.741 (2)°	*T* = 298 K
β = 84.056 (2)°	Block, blue
γ = 76.728 (1)°	0.18 × 0.14 × 0.08 mm
*V* = 702.84 (14) Å^3^	

### Data collection

**Table:**

Siemens SMART CCD diffractometer	2435 independent reflections
Radiation source: fine-focus sealed tube	2137 reflections with *I* > 2σ(*I*)
graphite	*R*_int_ = 0.018
ω scans	θ_max_ = 25.0°, θ_min_ = 1.8°
Absorption correction: multi-scan (*SADABS*; Sheldrick, 1996)	*h* = −7→8
*T*_min_ = 0.354, *T*_max_ = 0.594	*k* = −9→10
3669 measured reflections	*l* = −13→12

### Refinement

**Table:**

Refinement on *F*^2^	Primary atom site location: structure-invariant direct methods
Least-squares matrix: full	Secondary atom site location: difference Fourier map
*R*[*F*^2^ > 2σ(*F*^2^)] = 0.032	Hydrogen site location: inferred from neighbouring sites
*wR*(*F*^2^) = 0.082	H-atom parameters constrained
*S* = 1.02	*w* = 1/[σ^2^(*F*_o_^2^) + (0.0433*P*)^2^ + 0.8458*P*] where *P* = (*F*_o_^2^ + 2*F*_c_^2^)/3
2435 reflections	(Δ/σ)_max_ = 0.001
199 parameters	Δρ_max_ = 0.61 e Å^−^^3^
0 restraints	Δρ_min_ = −0.59 e Å^−^^3^

### Special details

**Table:**

Geometry. All esds (except the esd in the dihedral angle between two l.s. planes) are estimated using the full covariance matrix. The cell esds are taken into account individually in the estimation of esds in distances, angles and torsion angles; correlations between esds in cell parameters are only used when they are defined by crystal symmetry. An approximate (isotropic) treatment of cell esds is used for estimating esds involving l.s. planes.
Refinement. Refinement of F^2^ against ALL reflections. The weighted R-factor wR and goodness of fit S are based on F^2^, conventional R-factors R are based on F, with F set to zero for negative F^2^. The threshold expression of F^2^ > 2sigma(F^2^) is used only for calculating R-factors(gt) etc. and is not relevant to the choice of reflections for refinement. R-factors based on F^2^ are statistically about twice as large as those based on F, and R- factors based on ALL data will be even larger.

### Fractional atomic coordinates and isotropic or equivalent isotropic displacement parameters (Å^2^)

**Table:**

	*x*	*y*	*z*	*U*_iso_*/*U*_eq_	
Cu1	0.36391 (7)	0.20148 (5)	0.83934 (4)	0.02864 (15)	
Br1	0.09458 (7)	0.53067 (5)	0.65643 (4)	0.04093 (15)	
Br2	0.66479 (7)	0.36146 (5)	0.64489 (4)	0.04116 (15)	
N1	0.2408 (5)	0.4351 (4)	0.8722 (3)	0.0258 (7)	
N2	0.3743 (5)	0.1983 (3)	0.6656 (3)	0.0256 (7)	
O1	0.3617 (5)	0.1928 (3)	1.0067 (2)	0.0382 (7)	
O2	0.3544 (5)	0.3296 (4)	1.1627 (3)	0.0485 (8)	
O3	0.1337 (4)	0.0956 (4)	0.8258 (3)	0.0382 (7)	
O4	−0.0273 (4)	0.0169 (3)	0.6914 (3)	0.0383 (7)	
O5	0.6281 (5)	0.0513 (4)	0.8380 (3)	0.0558 (10)	
H5A	0.6454	−0.0203	0.8893	0.067*	
H5B	0.7377	0.0508	0.7972	0.067*	
C1	0.3350 (6)	0.3182 (5)	1.0602 (3)	0.0320 (9)	
C2	0.2678 (5)	0.4583 (5)	0.9843 (3)	0.0283 (9)	
C3	0.2265 (6)	0.6001 (5)	1.0303 (4)	0.0379 (10)	
H3	0.2459	0.6118	1.1081	0.046*	
C4	0.1558 (7)	0.7247 (5)	0.9590 (4)	0.0417 (11)	
H4	0.1324	0.8213	0.9871	0.050*	
C5	0.1210 (6)	0.7029 (5)	0.8466 (4)	0.0366 (10)	
H5	0.0703	0.7839	0.7973	0.044*	
C6	0.1633 (6)	0.5563 (5)	0.8081 (4)	0.0294 (9)	
C7	0.1021 (6)	0.0759 (4)	0.7223 (3)	0.0281 (9)	
C8	0.2405 (6)	0.1316 (4)	0.6261 (3)	0.0276 (8)	
C9	0.2250 (6)	0.1216 (5)	0.5081 (4)	0.0346 (10)	
H9	0.1292	0.0773	0.4831	0.042*	
C10	0.3536 (7)	0.1783 (5)	0.4273 (4)	0.0379 (10)	
H10	0.3463	0.1712	0.3472	0.045*	
C11	0.4922 (7)	0.2450 (5)	0.4658 (4)	0.0364 (10)	
H11	0.5812	0.2827	0.4128	0.044*	
C12	0.4961 (6)	0.2548 (4)	0.5859 (3)	0.0291 (9)	

### Atomic displacement parameters (Å^2^)

**Table:**

	*U*^11^	*U*^22^	*U*^33^	*U*^12^	*U*^13^	*U*^23^
Cu1	0.0328 (3)	0.0302 (3)	0.0214 (3)	−0.0060 (2)	−0.00068 (19)	0.0041 (2)
Br1	0.0450 (3)	0.0414 (3)	0.0322 (2)	−0.0002 (2)	−0.00934 (19)	0.00599 (19)
Br2	0.0424 (3)	0.0467 (3)	0.0401 (3)	−0.0234 (2)	−0.00604 (19)	0.0080 (2)
N1	0.0249 (17)	0.0264 (17)	0.0248 (17)	−0.0051 (14)	0.0008 (13)	0.0021 (13)
N2	0.0277 (17)	0.0233 (16)	0.0234 (16)	−0.0040 (13)	0.0016 (13)	0.0044 (13)
O1	0.0517 (19)	0.0355 (17)	0.0216 (14)	−0.0001 (14)	−0.0030 (13)	0.0073 (12)
O2	0.057 (2)	0.066 (2)	0.0218 (16)	−0.0124 (17)	−0.0057 (14)	0.0033 (15)
O3	0.0424 (18)	0.0476 (18)	0.0292 (16)	−0.0242 (14)	0.0046 (13)	0.0029 (13)
O4	0.0373 (17)	0.0439 (18)	0.0385 (17)	−0.0202 (14)	−0.0019 (13)	−0.0003 (14)
O5	0.047 (2)	0.055 (2)	0.045 (2)	0.0162 (17)	0.0127 (16)	0.0257 (17)
C1	0.025 (2)	0.044 (3)	0.026 (2)	−0.0064 (18)	0.0013 (16)	0.0013 (18)
C2	0.0190 (19)	0.037 (2)	0.028 (2)	−0.0062 (16)	0.0037 (15)	−0.0031 (17)
C3	0.034 (2)	0.045 (3)	0.037 (2)	−0.012 (2)	0.0014 (18)	−0.012 (2)
C4	0.038 (3)	0.035 (2)	0.054 (3)	−0.012 (2)	0.004 (2)	−0.010 (2)
C5	0.030 (2)	0.027 (2)	0.049 (3)	−0.0039 (18)	0.0033 (19)	0.0047 (19)
C6	0.0221 (19)	0.036 (2)	0.029 (2)	−0.0073 (17)	0.0015 (16)	0.0054 (17)
C7	0.031 (2)	0.023 (2)	0.029 (2)	−0.0046 (16)	0.0003 (16)	0.0024 (16)
C8	0.031 (2)	0.0210 (19)	0.029 (2)	−0.0027 (16)	−0.0025 (16)	0.0045 (16)
C9	0.041 (2)	0.029 (2)	0.034 (2)	−0.0060 (19)	−0.0069 (19)	−0.0024 (18)
C10	0.052 (3)	0.040 (3)	0.021 (2)	−0.012 (2)	−0.0041 (19)	0.0040 (18)
C11	0.045 (3)	0.032 (2)	0.027 (2)	−0.0048 (19)	0.0056 (18)	0.0056 (18)
C12	0.028 (2)	0.028 (2)	0.029 (2)	−0.0033 (17)	−0.0007 (16)	0.0046 (16)

### Geometric parameters (Å, °)

**Table:**

Cu1—O1	1.912 (3)	C1—C2	1.514 (6)
Cu1—N2	1.985 (3)	C2—C3	1.384 (6)
Cu1—O5	2.022 (3)	C3—C4	1.387 (6)
Cu1—O3	2.072 (3)	C3—H3	0.9300
Cu1—N1	2.148 (3)	C4—C5	1.367 (7)
Br1—C6	1.889 (4)	C4—H4	0.9300
Br2—C12	1.882 (4)	C5—C6	1.391 (6)
N1—C6	1.330 (5)	C5—H5	0.9300
N1—C2	1.351 (5)	C7—C8	1.529 (6)
N2—C12	1.342 (5)	C8—C9	1.377 (6)
N2—C8	1.348 (5)	C9—C10	1.382 (6)
O1—C1	1.296 (5)	C9—H9	0.9300
O2—C1	1.208 (5)	C10—C11	1.371 (6)
O3—C7	1.257 (5)	C10—H10	0.9300
O4—C7	1.239 (5)	C11—C12	1.387 (6)
O5—H5A	0.8499	C11—H11	0.9300
O5—H5B	0.8499		
			
O1—Cu1—N2	176.76 (12)	C4—C3—H3	120.4
O1—Cu1—O5	86.47 (13)	C5—C4—C3	118.8 (4)
N2—Cu1—O5	90.80 (13)	C5—C4—H4	120.6
O1—Cu1—O3	98.49 (13)	C3—C4—H4	120.6
N2—Cu1—O3	80.87 (13)	C4—C5—C6	118.2 (4)
O5—Cu1—O3	111.31 (14)	C4—C5—H5	120.9
O1—Cu1—N1	81.11 (12)	C6—C5—H5	120.9
N2—Cu1—N1	102.11 (12)	N1—C6—C5	124.4 (4)
O5—Cu1—N1	139.28 (14)	N1—C6—Br1	118.9 (3)
O3—Cu1—N1	108.83 (12)	C5—C6—Br1	116.6 (3)
C6—N1—C2	116.5 (3)	O4—C7—O3	126.9 (4)
C6—N1—Cu1	135.6 (3)	O4—C7—C8	117.7 (3)
C2—N1—Cu1	107.6 (2)	O3—C7—C8	115.3 (4)
C12—N2—C8	118.0 (3)	N2—C8—C9	122.1 (4)
C12—N2—Cu1	127.6 (3)	N2—C8—C7	114.7 (3)
C8—N2—Cu1	114.4 (3)	C9—C8—C7	123.2 (4)
C1—O1—Cu1	118.1 (3)	C8—C9—C10	119.1 (4)
C7—O3—Cu1	114.7 (3)	C8—C9—H9	120.4
Cu1—O5—H5A	120.1	C10—C9—H9	120.4
Cu1—O5—H5B	130.5	C11—C10—C9	119.6 (4)
H5A—O5—H5B	109.1	C11—C10—H10	120.2
O2—C1—O1	125.5 (4)	C9—C10—H10	120.2
O2—C1—C2	119.8 (4)	C10—C11—C12	118.2 (4)
O1—C1—C2	114.6 (3)	C10—C11—H11	120.9
N1—C2—C3	122.8 (4)	C12—C11—H11	120.9
N1—C2—C1	116.0 (3)	N2—C12—C11	122.9 (4)
C3—C2—C1	121.2 (4)	N2—C12—Br2	116.4 (3)
C2—C3—C4	119.1 (4)	C11—C12—Br2	120.5 (3)
C2—C3—H3	120.4		
			
O1—Cu1—N1—C6	−172.9 (4)	O2—C1—C2—C3	−0.5 (6)
N2—Cu1—N1—C6	7.5 (4)	O1—C1—C2—C3	177.7 (4)
O5—Cu1—N1—C6	113.2 (4)	N1—C2—C3—C4	−0.5 (6)
O3—Cu1—N1—C6	−76.9 (4)	C1—C2—C3—C4	−176.8 (4)
O1—Cu1—N1—C2	13.3 (3)	C2—C3—C4—C5	2.8 (6)
N2—Cu1—N1—C2	−166.3 (2)	C3—C4—C5—C6	−1.7 (6)
O5—Cu1—N1—C2	−60.6 (3)	C2—N1—C6—C5	4.0 (6)
O3—Cu1—N1—C2	109.3 (2)	Cu1—N1—C6—C5	−169.3 (3)
O1—Cu1—N2—C12	−103 (2)	C2—N1—C6—Br1	−173.3 (3)
O5—Cu1—N2—C12	−70.2 (3)	Cu1—N1—C6—Br1	13.4 (5)
O3—Cu1—N2—C12	178.3 (3)	C4—C5—C6—N1	−1.9 (6)
N1—Cu1—N2—C12	70.9 (3)	C4—C5—C6—Br1	175.5 (3)
O1—Cu1—N2—C8	78 (2)	Cu1—O3—C7—O4	−179.4 (3)
O5—Cu1—N2—C8	110.3 (3)	Cu1—O3—C7—C8	0.8 (4)
O3—Cu1—N2—C8	−1.2 (3)	C12—N2—C8—C9	−0.6 (5)
N1—Cu1—N2—C8	−108.6 (3)	Cu1—N2—C8—C9	179.0 (3)
N2—Cu1—O1—C1	160 (2)	C12—N2—C8—C7	−177.7 (3)
O5—Cu1—O1—C1	127.0 (3)	Cu1—N2—C8—C7	1.9 (4)
O3—Cu1—O1—C1	−122.0 (3)	O4—C7—C8—N2	178.3 (3)
N1—Cu1—O1—C1	−14.1 (3)	O3—C7—C8—N2	−1.8 (5)
O1—Cu1—O3—C7	−176.6 (3)	O4—C7—C8—C9	1.3 (6)
N2—Cu1—O3—C7	0.2 (3)	O3—C7—C8—C9	−178.8 (4)
O5—Cu1—O3—C7	−87.1 (3)	N2—C8—C9—C10	1.6 (6)
N1—Cu1—O3—C7	100.0 (3)	C7—C8—C9—C10	178.4 (4)
Cu1—O1—C1—O2	−170.2 (4)	C8—C9—C10—C11	−0.9 (6)
Cu1—O1—C1—C2	11.7 (5)	C9—C10—C11—C12	−0.8 (6)
C6—N1—C2—C3	−2.8 (6)	C8—N2—C12—C11	−1.2 (6)
Cu1—N1—C2—C3	172.3 (3)	Cu1—N2—C12—C11	179.3 (3)
C6—N1—C2—C1	173.6 (3)	C8—N2—C12—Br2	174.8 (3)
Cu1—N1—C2—C1	−11.2 (4)	Cu1—N2—C12—Br2	−4.7 (4)
O2—C1—C2—N1	−176.9 (4)	C10—C11—C12—N2	1.9 (6)
O1—C1—C2—N1	1.2 (5)	C10—C11—C12—Br2	−174.0 (3)

### Hydrogen-bond geometry (Å, °)

**Table:**

*D*—H···*A*	*D*—H	H···*A*	*D*···*A*	*D*—H···*A*
O5—H5A···O1^i^	0.85	1.93	2.765 (4)	168
O5—H5B···O4^ii^	0.85	1.90	2.743 (4)	169

Symmetry codes: (i) −*x*+1, −*y*, −*z*+2; (ii) *x*+1, *y*, *z*.
